# Development of entrustable professional activities framework for clinical microbiology residency: a national multi-step consensus using modified Delphi study

**DOI:** 10.1186/s12909-025-07345-x

**Published:** 2025-05-22

**Authors:** Mennatallah H. Rizk, Niveen Ghoraba, Hanaa S. Elhoshy, Omayma Hamed

**Affiliations:** 1 Medical Education Department, Armed Force Colleague of Medicine (AFCM), Cairo, Egypt; 2 Medical Education Department, Faculty of Medicine, Alexandria University, Alexandria, Egypt; 3 Clinical Pathology Department Faculty of Medicine, Al-Azhar University for Girls, Cairo, Egypt

**Keywords:** Professional competence, Microbiology, Education, Medical, Graduate, Delphi technique, Validation studies, Competency-Based education

## Abstract

**Background:**

While competency-based education has gained prominence in preparing professionals for practice, clinical microbiology residency programs face a challenge in defining specific, observable tasks that align with Entrustable Professional Activities (EPAs). The current lack of a standardized set of EPAs tailored to clinical microbiology creates a gap in assessing learner proficiency and educational outcomes.

**Objectives:**

This study aims to develop and validate a set of specific EPAs for clinical microbiology using a multi-step national expert consensus-building process.

**Methods:**

This study was conducted in Egypt, involving experts from various medical schools across the country. As the first step, a thorough literature review was undertaken to identify potential EPAs pertinent to clinical microbiology residency programs. Then, evaluation of EPAs for quality and structure using EQual rubric involved five experts in medical education and clinical microbiology, resulting in the confirmation of relevant EPAs. Subsequently, three rounds of the modified Delphi method were employed, engaging ten clinical microbiology experts from various medical schools. Simultaneously, content validity was assessed based on these ratings. Participants also determined the appropriate year of entrustment for each EPA item, and an 80% Validity index agreement threshold was calculated to ensure consensus among participant groups.

**Results:**

The use of the literature review and initial expert evaluation using EQual rubric confirmed 39 out of the initially identified 43 EPAs. Following the modified Delphi method rounds, 16 EPAs gained acceptance, signifying their relevance and appropriateness for clinical microbiology residency training. These EPAs were categorized into key areas, including preanalytical testing and quality assurance, microbiological techniques and diagnostics, infection control and safety practices, clinical leadership and teamwork, research and development, and laboratory management and communication.

**Conclusions:**

This study developed 16 EPAs for clinical microbiology residency programs. These EPAs were developed using a robust multi step validation study. This provides a further step towards competency-based postgraduate training in clinical microbiology.

**Clinical trial number:**

Not applicable.

**Supplementary Information:**

The online version contains supplementary material available at 10.1186/s12909-025-07345-x.

## Introduction

The concept of entrustability of professional activities, introduced in 2005, marked a paradigm shift in medical education. Rather than assessing individual skills, EPAs focus on whether trainees can be entrusted with specific professional activities that integrate multiple competencies [1] The latest definition of EPA as rehearsed by Ten Cate in 2021 is an EPA is a unit of professional practice that can be fully entrusted to a trainee, once he or she has demonstrated the necessary competence to execute this activity unsupervised. EPAs have enabled the shift towards competency-based medical education (CBME) by providing a structured approach to evaluate whether trainees can perform integrated, authentic tasks independently, thereby bridging the gap between theoretical knowledge and practical application [2]. The development of EPAs frameworks in health care professions has been extensive and varied across different specialties [3]. By defining specific activities that trainees must be able to perform independently, EPAs create a structured approach to assess and develop essential skills in real-world settings. This operationalization ensures that the training aligns closely with professional practice, facilitating a more effective transition from education to clinical responsibilities [4]. These frameworks ensure an authentic and systematic learning process by integrating competencies into practical, real-world settings. Accordingly, it guarantees that workplace learning, and assessment are not only comprehensive but also aligned with professional standards and expectations, leading to development of a more competent healthcare workforce [5].

The validation process of EPAs framework for each specialty is a crucial step to guarantee that the EPAs thoroughly represent the authentic and relevant tasks required in that field. This process involves a rigorous assessment by stakeholders and subject matter experts using Ten Cate’s published EPAs features and guidelines [2, 6]. The main aim of validation process is to confirm that the EPAs are not only aligned with the specific competencies needed but also reflect the real-world duties and challenges managed by health care professionals in that specialty. There are several approaches outlined in the literature for validating EPAs among experts. These include expert meetings (both national and international), surveys, the Delphi procedure, the nominal group technique, and interviews [4, 7].

The Delphi procedure is one of the commonly used approaches for validating EPAs [8]. A quick search in PubMed (July 2024) yields over 100 journal articles since 2013 with the word “(Entrustable Professional Activities) AND (Delphi) in their title or abstract, with a rapid increase since 2020. This method is widely recognized for its effectiveness in gathering expert opinions and establishing consensus on complex issues that benefit from collective subjective judgments rather than analytical solutions [9]. The Delphi procedure allowed for engagement of a diverse group of experts without requiring in-person meetings, thereby facilitating participation from various geographical locations and healthcare settings. By ensuring anonymity throughout the Delphi process, it mitigated potential biases and encouraged experts to provide unbiased input without being influenced by other participants [10, 11]. Similar Delphi methodologies have been successfully employed to develop EPA frameworks in various health professions education programs [8, 12–17].

Numerous EPAs frameworks have been developed across many medical specialties. However, as the adoption of EPAs has expanded, concerns about their quality, relevance, and applicability have also grown. Consequently, there is an urgent need to meticulously evaluate existing EPAs to ensure they meet the intended educational outcomes and maintain high standards of clinical training. This evaluation is critical not only for revising and refining EPAs but also for validating their effectiveness in fostering competent and dependable healthcare professionals [3]. Tools such as the Quality of Entrustable Professional Activities (QUEPA) and the Queen’s EPA Quality Rubric (EQual) have been developed to facilitate the systematic evaluation and refinement of EPAs [18, 19]. The QUEPA tool includes 15 items with excellent reliability, categorized into four distinct factors: Realistic and Generalizable (6 items), Observable (3 items), Focused (3 items), and Multiple Competencies (3 items). The QUEPA tool has been criticized for not being completely aligned with the defining qualities of EPAs [18]. Moreover, it uses normative scales for each item without descriptive anchors. Also, it does not provide predefined cut scores for acceptability of an EPA’s quality, restricting its utility and interpretation [4, 6, 18]. The EQual rubric overcomes these critiques as it is criterion based and based on the defining features of EPAs as well as cutoff scores that were determined for each domain and the overall scores. It is composed of 14 questions which evaluates the 3 domains of EPAs: EPAs as discrete units of work; EPAs as entrustable, essential, and important tasks of the profession; and EPAs’ curricular role [18, 20].

Clinical microbiology residency training in Egypt is structured to equip future practitioners with the skills necessary to address complex microbiological challenges. Typically lasting three years, these programs combine theoretical knowledge with practical experience in university-affiliated hospitals and major teaching institutions. However, postgraduate curricula often lack standardization and do not comprehensively address the specific competencies required in this specialty Based on recent literature, there is a notable absence of EPAs tailored for clinical microbiology residency training. This gap highlights significant challenges faced by residency programs, including inadequate focus on critical areas such as diagnostic accuracy, infection control practices, and laboratory management skills. These deficiencies hinder residents’ preparedness to tackle complex healthcare issues, such as antimicrobial resistance and emerging infectious diseases [21–24]. Therefore, our study objective is to establish EPAs specifically designed for clinical microbiology training by achieving consensus among clinical educators and experts in the field. These EPAs will be integrated into the residency curriculum to ensure comprehensive training and assessment of residents’ competencies. The developed EPAs framework will provide the necessary tools for clinical educators to effectively assess trainees’ readiness for independent practice. This initiative aims to enhance healthcare services by developing a competent workforce trained through a structured EPA approach.

## Methods

### Research design

We used an exploratory sequential mixed methods design to develop and validate a set of EPAs for clinical microbiology residency training in Egypt. The aim was to establish a framework of EPAs that are relevant, clear, and aligned with the needs of local training programs. The study took place in Egypt from May through December 2023. The exploratory sequential mixed methods design was chosen to allow for a comprehensive approach, starting with qualitative exploration to identify relevant EPAs, followed by quantitative validation through a modified Delphi technique to achieve consensus among experts. This approach is particularly suitable when addressing complex educational interventions, where both expert opinion and structured evaluation are valuable.

### Participants

A purposive sampling approach was used to recruit clinical microbiology experts for the validation process. Experts were selected based on predefined inclusion and exclusion criteria to ensure a high level of expertise and relevance to the study objectives. Inclusion criteria required that candidates be clinical microbiologists with a minimum of five years of postgraduate experience, active involvement in residency training, and demonstrated contributions to the field through publications or presentations. Exclusion criteria included individuals holding administrative positions without current clinical practice involvement or those with postgraduate experience, less than 5 years. A total of 15 experts were initially invited by email to participate, with follow-up reminders sent over a period of 10 days. Ultimately, 10 experts (66.7% response rate) agreed to participate in the study, ensuring a diverse representation across various medical schools in Egypt.

### Development and validation of EPAs (Phases 1–3)

**Fig. 1:**
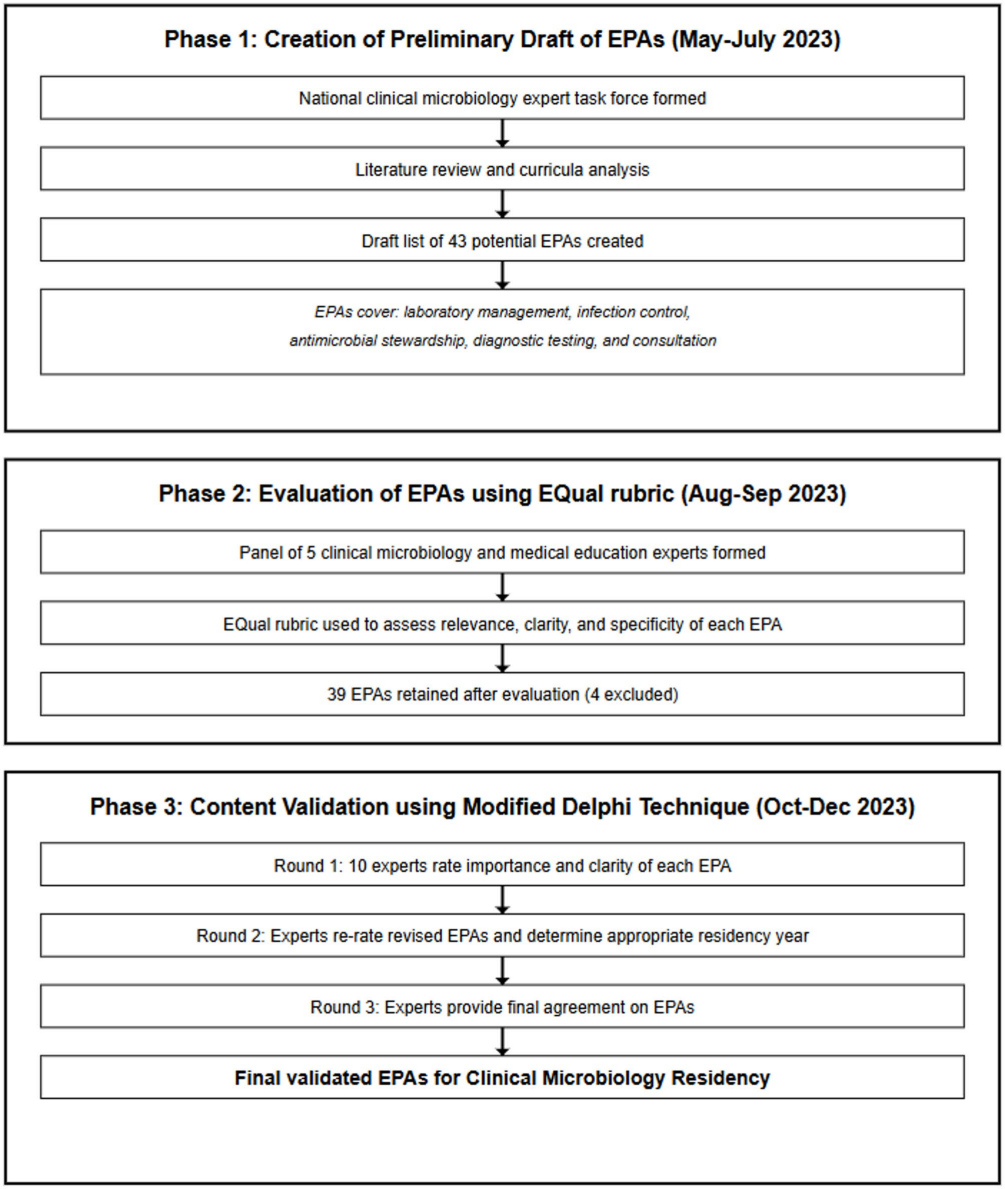
The three phases of development for the clinical microbiology EPAs framework

The development and validation of the EPAs were conducted in three phases: [1] creation of a preliminary draft of EPAs, through a qualitative process involving a clinical microbiology task force, which reviewed existing literature and guidelines to identify core competencies; [2] evaluation of EPAs using the EQual rubric, to assess their relevance, clarity, and specificity; and [3] content validation using the Modified Delphi technique, which involved experts rating the importance and clarity of each EPA and specifying the year of entrustment. Details of each phase are shown in Figure (1).

### Phase 1: creation of a preliminary draft of EPAs

In the first phase of our study, a comprehensive literature review was conducted to inform the development of the EPAs. We searched major databases such as PubMed, Scopus, and Google Scholar for relevant studies and guidelines related to clinical microbiology competencies. Key resources were the competency-based postgraduate curriculum of microbiology departments, ACGME Program Requirements for Graduate Medical Education in Hematopathology, Entrustable Professional Activities for Pathology developed by The Royal College of Pathologists [25–27],.

The development of the EPAs involved a combination of inductive and deductive approaches. Initially, a deductive approach was employed by analyzing existing frameworks and guidelines to identify core competencies in clinical microbiology. This involved systematically categorizing and synthesizing the information from these sources to create a preliminary list of EPAs. Subsequently, an inductive approach was applied through expert discussions. The task force (composed of 3 clinical microbiologists) was formed by inviting experts from various departments of clinical microbiology at public medical schools. These experts were chosen based on their academic and clinical experience in microbiology, as well as their involvement in residency training programs. The task force played a crucial role in developing the initial list of potential EPAs through a qualitative process involving literature review and expert judgments.

### Phase 2: evaluation of EPAs using equal rubric

The preliminary EPAs were evaluated by a panel of five experts (3 clinical microbiology and 2 medical education). The EQual rubric was used to assess the relevance, clarity, and specificity of each EPA [18]. Each expert panel member utilized a separate Google Sheet to independently input their ratings for the EPAs. This approach allowed for individual assessments of relevance, clarity, and specificity without influence from other panellists. The Google Sheets were structured with predefined criteria and anchored Likert scales to standardize the evaluation process. After completing their evaluations, the individual scores were aggregated to derive an overall rating for each EPA. This method not only facilitated efficient data collection but also ensured transparency and anonymity in the evaluation process. The modified Angoff approach used by Taylor and colleagues to establish an overall cutoff score of 4.07 for the EQual rubric indicates that any EPA scoring below this threshold is considered insufficient in key domains and may require revision [18, 20].

### Phase 3: content validation using modified delphi technique

We utilized a modified Delphi method to achieve consensus among clinical microbiology experts regarding the importance, clarity, and entrustment levels of the EPAs. This approach was chosen for its flexibility and ability to adapt to our study’s specific needs, while maintaining the core principles of expert opinion and iterative refinement [28]. Our modified Delphi process involved several key adjustments to the traditional method. First, we provided structured feedback to participants after each round, including aggregated results and qualitative comments. This allowed experts to reflect on the broader group’s perspectives and adjust their ratings accordingly. Second, we limited the process to three rounds, which was sufficient to achieve stability in the ratings and consensus among experts. Finally, each round had focused objectives, such as rating importance and clarity in Round 1, determining entrustment years in Round 2, and finalizing agreement in Round 3. To ensure clarity and consistency throughout the study, we maintained the same numbering for EPAs across all Delphi rounds. This deliberate choice prevented any potential confusion that might arise from renumbering or reordering EPAs between rounds and facilitated tracking the evolution of each EPA throughout the process.

#### Round 1

The expert panel composed of ten clinical microbiology experts rated the importance and clarity of each EPA using a 5-point Likert scale along with an open text box for comments. They rated the importance of each EPA using a Likert scale from 1 to 5 (1 = none, 2 = weak, 3 = moderate, 4 = high and 5 = very high). They also rated the clarity of each EPA’s description using a Likert scale from 1 to 5 (1 = very poor, 2 = poor, 3 = neither bad nor good, 4 = good, and 5 = very good). (APPENDIX 2)

#### Round 2

Experts received a summary of the results from Round 1, which included aggregated ratings for each EPA and anonymized qualitative feedback provided by the panel. This allowed experts to reflect on their initial evaluations considering the broader group’s input. Additionally, revised EPA descriptions were shared, incorporating any modifications based on Round 1 feedback. Experts were then asked to re-rate the importance and clarity of each EPA and assign the appropriate year of residency for entrustment without direct supervision. (APPENDIX 3)

#### Round 3

In this final round, experts were provided with the revised EPAs based on Round 2 feedback, along with a summary of the aggregated results from Round 2, including mean scores and any changes made to the EPAs. They were asked to review these updates and provide their agreement (yes or no) with the final list of EPAs. This step ensured consensus on the finalized framework. (APPENDIX 4)

### Data analysis

In our study, the data from each Delphi round were analysed using both quantitative and qualitative methods. For each round, we calculated the Content Validity Index (CVI) for each EPA by dividing the number of experts who rated an EPA with a score of 4 or 5 by the total number of experts participating in that round. A CVI of 0.8 or higher indicated sufficient content validity, while scores between 0.70 and 0.79 suggested that the item required revision, and scores below 0.70 indicated that the corresponding EPA should be eliminated [29].

Additionally, we calculated the mean ratings and standard deviations for each EPA based on expert evaluations to assess consensus and variability in scores. The Intra-class Correlation (ICC) was also computed to evaluate inter-rater reliability across rounds. ICC estimates and their 95% confidence intervals were calculated based on a mean rating, absolute agreement, and 2-way mixed-effects model. Interpretation of ICC Values as: ICC < 0.40 means poor correlation, 0.40 ≤ ICC < 0.59 means fair correlation, 0.60 ≤ ICC < 0.74 means good correlation, 0.75 ≤ ICC ≤ 1.00 means excellent correlation [30].

Free-text answers were analyzed qualitatively using thematic analysis. Two research authors independently coded and categorized responses to identify common themes and patterns, ensuring consistency in coding.

## Results

This study is composed of three phases to develop and validate a set of Entrustable Professional Activities (EPAs) for clinical microbiology residency training. he results of each phase are presented below.

### Phase 1 results: creation of a preliminary draft of EPAs

The phase 1 of the study involved a review of existing literature, ACGME requirements, and existing EPAs by a task force of three clinical microbiologists. This process led to the generation of 43 potential EPAs covering a range of domains within clinical microbiology. These EPAs encompassed areas such as laboratory management, infection control, antimicrobial stewardship, diagnostic testing, and consultation. The process included in-depth discussions among the task force members to ensure the EPAs were relevant and comprehensive, reflecting the essential activities performed by clinical microbiologists in Egypt. (APPENDIX 1)

### Phase 2 results: evaluation of EPAs using EQual rubric

The 43 potential EPAs were then subjected to evaluation using the EQual rubric to assess their relevance, clarity, and specificity. A panel of five experts, including three clinical microbiologists and two medical education specialists, independently reviewed each EPA using the EQual rubric. Qualitative feedback from the EQual rubric was considered in the refinement process, though not formally analysed. The following EPAs were excluded based on EQual rubric evaluation:

EPA – 14 Perform various other serology techniques like ELISA, and IFA.

EPA – 20 Ensure compliance with relevant legislative and regulatory frameworks.

EPA – 42 Familiar with norms & requirements of NABL, NABH accreditation.

EPA – 43 Perform and validate quality control procedures for newly received culture media and reagents.

These EPAs scored below the predefined cutoff score of 4.07, indicating they did not meet the criteria for alignment with EPA standards. This resulted in a refined list of 39 EPAs that proceeded to the Delphi validation phase. (APPENDIX 1)

### The results of qualitative analysis for phases 1 and 2

The EPAs generated in Phases 1 and 2 (Appendix 1) underwent qualitative rephrasing to enhance their clarity, specificity, and relevance to the context of clinical microbiology practice in Egypt. For example, EPA-2, initially stated as ‘Perform & interpret various staining techniques,’ was revised in Phase 3 to become ‘Perform & interpret various staining techniques like gram staining, Acid-fast staining, negative staining, and special staining,’ providing greater detail and guiding the resident on what is expected. Similarly, EPA-15, initially ‘prepare a protocol for investigating any outbreak in the area’ was changed to ‘prepare a protocol for investigating any outbreak in the area like cholera, typhoid, brucellosis, and viral infections’, which is a more detailed. Another example is the EPA − 35 that initially mentioned ‘Compose a diagnostic report for clinical laboratory testing’, was rephrased to ‘Compose a diagnostic report for clinical laboratory testing requiring pathologist interpretation’. This EPA emphasizes that diagnostic reports are not just technical documents, and they require the ability to convey information. EPA – 36 Evaluate and report critical values in the clinical laboratory was rephrased to Identity, Evaluate, report, and communicate critical value and clinically urgent results. EPA – 37 Optimize test utilization (AP/CP laboratory management) was rephrased to EPA37: Optimize test utilization in the Clinical Microbiology Laboratory. These changes resulted in the development of EPAs used in phase 3.

### Phase 3 results: content validation using modified Delphi technique

Total number of clinical microbiology experts were 10 experts. All Experts who accepted to participate in the study were females. The experts represented five different public universities in Egypt. Table 1 shows the demographic data of expert panel.

**Table 1:** Demographic data of expert respondents of the three Delphi rounds

	Frequency	Relative Frequency
**Academic ranking**		
Professor	3	30%
Assistant professor	4	40%
Lecturer	2	20%
Assistant lecturer	1	10%
**Years of experience**		
≥ 5–10	1	10%
> 10–15	3	30%
> 15–20	3	30%
> 20	3	30%
**University**		
Al-Azhar University	5	50%
Ain Shams University	1	10%
Cairo University	2	20%
Alexandria university	1	10%
Zagazig University	1	10%

### Results of round 1 modified Delphi technique

In the first Delphi round, 10 clinical Microbiology experts carefully reviewed 39 EPAs derived from second phase of the study. The EPAs that had sufficient content validity with CVI of 0.8 or higher were 15 (38.4%), while EPAs that required revision with CVI within the range of 0.70 and 0.79 were 4 (10.3%), and those EPAs which required elimination with CVI below 0.70 were 20 (51.3%). A complete list of reviewed EPAs by experts in round 1 is shown in Table 2. The Intraclass Correlation Coefficient (ICC) analysis was performed to assess the reliability of the measurements. The results indicated that the Single Measures ICC was 0.216, with a 95% confidence interval ranging from 0.111 to 0.417, suggesting poor reliability for individual measurements. On the other hand, the Average Measures ICC was significantly higher at 0.839, with a 95% confidence interval between 0.703 and 0.931, indicating excellent reliability when averaging across multiple measurements. Both ICC values are statistically significant (*p* < 0.001), as evidenced by the F test results (Table 3).

**Table 2:** Content validity index (CVI) for of individual EPAs of round 1 modified Delphi technique

EPA	Experts in agreement	I-CVI:	Universal agreement (UA):	Next round validation
EPA1.	10	1	1	Qualified
EPA2.	10	1	1	Qualified
EPA3.	6	0.6	0	Not qualified
EPA4.	6	0.6	0	Not qualified
EPA5.	**10**	**1**	**1**	**Qualified**
EPA6.	**10**	**1**	**1**	**Qualified**
EPA7.	*7*	*0.7*	*0*	*Revision*
EPA8.	*7*	*0.7*	*0*	*Revision*
EPA9.	*7*	*0.7*	*0*	*Revision*
EPA10.	6	0.6	0	Not qualified
EPA11.	**9**	**0.9**	**0**	**Qualified**
EPA12.	**9**	**0.9**	**0**	**Qualified**
EPA13.	**10**	**1**	**1**	**Qualified**
EPA15.	4	0.4	0	Not qualified
EPA16.	**9**	**0.9**	**0**	**Qualified**
EPA17.	**8**	**0.8**	**0**	**Qualified**
EPA18.	3	0.3	0	Not qualified
EPA19.	**9**	**0.9**	**0**	**Qualified**
EPA21.	5	0.5	0	Not qualified
EPA22.	6	0.6	0	Not qualified
EPA23.	**8**	**0.8**	**0**	**Qualified**
EPA24.	4	0.4	0	Not qualified
EPA25.	4	0.4	0	Not qualified
EPA26.	6	0.6	0	Not qualified
EPA27.	5	0.5	0	Not qualified
EPA28.	3	0.3	0	Not qualified
EPA29.	*7*	*0.7*	*0*	*Revision*
EPA30.	**10**	**1**	**1**	**Qualified**
EPA31.	6	0.6	0	Not qualified
EPA32.	6	0.6	0	Not qualified
EPA33.	5	0.5	0	Not qualified
EPA34.	6	0.6	0	Not qualified
EPA35.	**9**	**0.9**	**0**	**Qualified**
EPA36.	**10**	**1**	**1**	**Qualified**
EPA37.	**9**	**0.9**	**0**	**Qualified**
EPA38.	4	0.4	0	Not qualified
EPA39.	5	0.5	0	Not qualified
EPA40.	4	0.4	0	Not qualified
EPA41.	3	0.3	0	Not qualified

**Table 3:** The intraclass correlation coefficient (ICC) for each (EPA) regarding the importance

	Intraclass Correlation^b^	95% Confidence Interval		F Test with True Value 0			
		Lower Bound	Upper Bound	Value	df1	df2	Sig
Single Measures	.216^a^	0.111	0.417	6.223	16	288	0.000
Average Measures	.839^c^	0.703	0.931	6.223	16	288	0.000

Two-way mixed effects model where people effects are random, and measures effects are fixed

a. The estimator is the same, whether the interaction effect is present or not

b. Type C intraclass correlation coefficients using a consistency definition. The between-measure variance is excluded from the denominator variance

c. This estimate is computed assuming the interaction effect is absent, because it is not estimable otherwise

### Results of round 2 modified Delphi technique

**Fig. 2:**
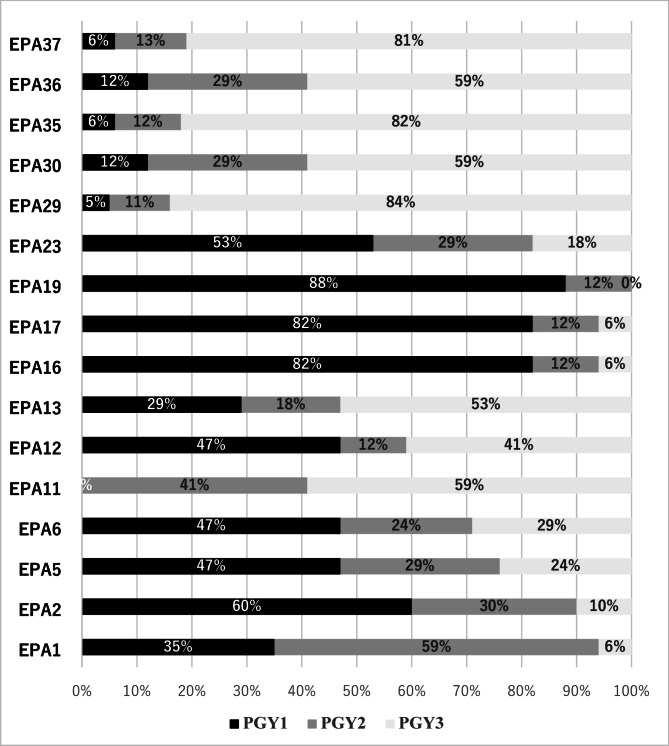
The final list of EPAs displayed by the year of entrustment

In the second Delphi round survey, the experts were asked first to review the **4** EPAs (EPAs 7, 8, 9, 29) which needed revision from round 1. Only **1** EPA (EPA29) was qualified for next round of validation and the other 3 EPAs were not qualified and eliminated. The experts also were asked to specify the year of entrustment (year of training, in which the EPA can be conducted without direct supervision) for each of the final **16** qualified EPAs. The years of entrustment were categorized as followed: First postgraduate year (PGY1), Second postgraduate year (PGY2), third postgraduate year (PGY3) as shown in Fig. 2. The Table 4 highlights that certain EPAs, such as EPA8, EPA9, and EPA10, are entrusted to residents in PGY1, with over 80% of experts agreeing that these activities can be performed independently in the first year. On the other hand, EPAs like EPA12, EPA14 and EPA16 are mostly entrusted during PGY3, with most experts indicating these activities are appropriate for independent practice in the final year of training. Additionally, EPAs with less than 80% consensus among experts, due to differences in training environments or individual resident progress. Such EPAs may require further discussion or could be entrusted on a case-by-case basis rather than as part of a standardized curriculum.

The Intraclass Correlation Coefficient (ICC) values presented in Table 5 indicate the reliability of measurements for each EPA over the years of entrustment. The Single Measures ICC of 0.140 (95% CI: 0.065 to 0.302) suggests poor reliability for individual measurements. However, the Average Measures ICC of 0.756 (95% CI: 0.570 to 0.892) indicates an excellent level of reliability when averaging multiple measurements. Both ICC values are statistically significant (*p* < 0.001), as evidenced by the F test results.

**Table 4:** The intraclass correlation coefficient (ICC) for each (EPA) regarding the year of entrustment

	Intraclass Correlation^b^	95% Confidence Interval		F Test with True Value 0			
		Lower Bound	Upper Bound	Value	df1	df2	Sig
Single Measures	.140^a^	0.065	0.302	6.223	16	288	0.000
Average Measures	.756^c^	0.570	0.892	6.223	16	288	0.000

Two-way mixed effects model where people effects are random, and measures effects are fixed

a. The estimator is the same, whether the interaction effect is present or not

b. Type A intraclass correlation coefficients using an absolute agreement definition

c. This estimate is computed assuming the interaction effect is absent, because it is not estimable otherwise

### Results of round 3 modified Delphi technique

In the third round, experts were asked to provide their agreement (Yes or No) about the final list of 16 EPAs which were qualified from the first and second rounds. The question that was asked for each EPA: This EPA is an essential component of practice, and the resident is expected to reach Level 4 (ready for unsupervised practice) in PGY… **Do You Agree**: **Yes…No**. Percentages of agreement (“yes” or “no”) were computed, and an 80% threshold was specified. Table (5) shows the level of agreement among experts about each EPA of the final list of EPAs. The final list of EPAs is presented in Appendix 5.

**Table 5:** The level of agreement among experts regarding the final list of EPAs in the third round of Delphi technique

EPAs	% of agreement (Answer: Yes)	Interpretation
EPA1: Provide guidance for the resolution of preanalytical testing issues.	**100%**	**Good agreement**
EPA2: Perform & interpret various staining techniques like gram staining. Acid-fast staining. negative staining and special staining	**91%**	**Good agreement**
EPA5: Carry out antibiotic sensitivity testing as per Current standard guidelines.	**91%**	**Good agreement**
EPA6: Identify the pathogenic bacteria by aerobic and anaerobic culture methods.	**91%**	**Good agreement**
EPA11: Able to manage needle stick injury documentation and care.	**91%**	**Good agreement**
EPA12: Instruct the technician for handling & disposal of biomedical wastes.	**91%**	**Good agreement**
EPA13: Aware & able to implement Infection control practices.	**100%**	**Good agreement**
EPA16: Carry out systematic research work.	**83%**	**Good agreement**
EPA17: Perform as a team worker/leader.	**91%**	**Good agreement**
EPA19: Advice on infection control measures	**91%**	**Good agreement**
EPA23: Implement, support, and develop procedures for safe laboratory practice.	**83%**	**Good agreement**
EPA29: Provide clinical leadership and support to the laboratory.	**91%**	**Good agreement**
EPA30: Use the laboratory service effectively in the investigation, diagnosis, and management of infection.	**100%**	**Good agreement**
EPA35: Compose a diagnostic report for clinical laboratory testing requiring pathologist interpretation.	**83%**	**Good agreement**
EPA36: Identity, Evaluate, report, and communicate critical value and clinically urgent results.	**91%**	**Good agreement**
EPA37: Optimize test utilization in the Clinical Microbiology Laboratory	**83%**	**Good agreement**

## Discussion

The development of EPAs framework for clinical microbiology residency programs marks a significant milestone in standardizing competency-based education in this field. Our study identified and validated 16 EPA items, addressing a critical gap in assessing residents’ proficiency and educational outcomes within clinical microbiology residency training. The validated EPAs not only provide a structured approach to clinical microbiology training but also offer a framework for improving educational outcomes. By focusing on essential activities that define the profession, these EPAs ensure that residents are equipped with the necessary competencies to excel in real-world clinical settings [4]. For instance, the inclusion of EPAs related to antimicrobial stewardship and infection control highlights the importance of these skills in contemporary clinical practice.

The initial list of 43 EPAs was refined to a final set of 16 through a rigorous evaluation process. Beyond the CVI scores, several factors influenced the elimination or modification of EPAs. Redundancy was a key consideration; if two EPAs covered similar competencies, the less specific or less critical one was removed to streamline the list and focus on essential activities. EPAs were modified if they lacked clarity or specificity. Feedback from the expert panel highlighted areas where descriptions needed refinement to ensure they were actionable and measurable. This process involved revising the language to make the EPAs more concise and relevant to real-world clinical practice. The modified Delphi process allowed for iterative feedback and consensus-building among experts. EPAs that did not align with the majority’s expectations or were considered less critical were either modified or excluded. This ensured that the final set of EPAs reflected a broad consensus among clinical microbiology experts. Additionally, EPAs were evaluated based on their clinical relevance and feasibility in real-world settings. Those that were deemed impractical or not commonly encountered in clinical practice were either refined or removed.

The use of a multi-step validation process to develop EPAs framework for a speciality is crucial for several reasons [31]. Firstly, it enhances the content validity of the EPAs by triangulating diverse expert perspectives. As noted by Ten Cate et al. (2015), EPAs should be carefully designed to represent the essential activities that define a profession [4]. By integrating a comprehensive literature review, EPAs evaluation by experts using the EQual rubric, and a modified Delphi method, our study ensures that the developed EPAs are both comprehensive and relevant to current practice. Secondly, the multi-step validation process allows for iterative refinement of the EPAs, addressing inconsistencies that may not be evident in a single-step validation process. This aligns with the recommendations of Touchie and Ten Cate (2016), who emphasize the importance of ongoing revision and improvement of EPAs to maintain their relevance and effectiveness [32].

The use of the EQual rubric in our validation process is significant. This tool, developed by Taylor et al. (2017), provides a structured framework for evaluating the quality of EPAs across multiple domains [18]. By including this rubric, we ensured that our EPAs meet established quality criteria, enhancing their reliability and applicability. The EQual rubric has been increasingly adopted in various medical specialties for EPAs evaluation [17, 20, 33]. Soran et al. (2020) used the EQual rubric to evaluate EPAs for internal medicine residency programs. Their study involved a multi-step process, initial EPA development through literature review and expert consensus, then evaluation of the developed EPAs using the EQual rubric by two study authors [34].

Our study used the modified Delphi method to enhance the construct validity of the EPAs by drawing on the collective expertise of field professionals. This consensus-building approach, as described by Hsu and Sandford (2007), is important in areas where professional judgment plays a significant role in defining professional skills [35]. Several studies reported the use of this method for development of EPAs frameworks either independently and in combination with other tools or methodologies [8, 14, 16, 17, 36].

As part of the validation process, we measured the inter-rater reliability as it is crucial to ensure highly consistent ratings. Intra-class correlation coefficient (ICC) was calculated to measure consistency of experts’ ratings on the year of entrustment of each EPA and the importance of each EPA. Intraclass correlation coefficient with a 95% confidence interval of round 1 of modified Delphi was 0.83 (where values between 0.70 and 0.93 which signify excellent correlation) and round 2 of modified Delphi was 0.756 (where values between 0.570 and 0.892 signify excellent correlation) [30]. Our findings are similar to many studies which reported ICC as a measure of reliability [19, 36, 37].

The distribution of EPAs across postgraduate years is crucial for ensuring that residents progressively develop the necessary competencies. Comparing this distribution with other clinical trainings, such as internal medicine or surgery, reveals both similarities and differences. For example, while all specialties require foundational competencies like patient assessment and management, clinical microbiology places a unique emphasis on laboratory diagnostics and antimicrobial therapy [34]. This comparison underscores the need for tailored EPA frameworks that reflect the specific demands of each specialty.

Handling EPAs with low consensus among experts is crucial for ensuring that competency-based education frameworks remain effective and relevant. Differences in training environments or individual resident progress may contribute to variations in expert opinions regarding certain EPAs, influencing how competencies are perceived and assessed across different settings [38]. EPAs with low consensus may require case-by-case entrustment decisions rather than inclusion in a standardized curriculum, allowing for flexibility and adaptation to specific learning contexts [34, 39]. This approach highlights the importance of flexibility in curriculum design, as educational programs must adapt EPAs based on local training conditions and individual learner needs. Ensuring that EPAs are socially valid and accepted by stakeholders is also crucial, involving assessments of their perceived acceptability and importance among target populations [39, 40].

The lower agreement scores for certain EPAs in our study highlight areas where expert opinions may vary due to differences in training environments or individual resident progress. This variability can be addressed by considering the role of elective EPAs, which are not universally required but can be offered to trainees who demonstrate advanced proficiency or special interests. Elective EPAs can include specialized procedures or skills that are not core to every graduate’s training but are valuable for those pursuing specific career paths (e.g., rare procedures only performed in specialized centres). By incorporating elective EPAs into the curriculum, programs can accommodate to the diverse needs and interests of residents while maintaining a focus on core competencies. This approach requires flexibility in curriculum design, allowing for personalized learning pathways that accommodate both core and elective EPAs [41, 42]. However, implementing such flexibility may pose challenges in authorities with rigid training structures, underscoring the need for adaptable educational frameworks that balance core requirements with opportunities for specialized training [43].

Implementing the EPA framework in clinical microbiology training requires careful consideration of practical applications. Medical educators can integrate these EPAs into curriculum design by mapping them onto specific learning objectives and assessment tools. For instance, EPAs related to laboratory diagnostics could be linked to hands-on training sessions and competency assessments [32]. In terms of alignment with existing competency frameworks, while our study did not specifically investigate frameworks in Egypt, the general approach to integrating EPAs into competency-based models can be applied. EPAs can be linked to broader competency domains and milestones, similar to how they are used in other specialties like emergency medicine and nursing education [44, 45] As medical education in Egypt moves towards competency-based models, our EPAs could potentially align with such frameworks by focusing on essential professional activities that define clinical microbiology practice.

### Limitations of the study

The study relied on a small sample size of experts in medical education and clinical microbiology for the initial EPA evaluation and validation process. While this was sufficient for a national consensus within Egypt, it may limit the generalizability of the identified EPAs to other countries with different educational systems and training requirements. The practice of clinical microbiology varies across countries, which could impact the applicability of these EPAs in other contexts. The study did not apply the identified EPAs in real-world clinical microbiology residency programs, which limits the assessment of their practical applicability and effectiveness in evaluating learner proficiency and educational outcomes.

### Future studies

Future studies could focus on the practical implementation of the identified EPAs within clinical microbiology residency programs. This includes assessing how these EPAs are integrated into existing curricula and evaluating their impact on resident performance and educational outcomes. Also, gathering feedback from various stakeholders, including residents, faculty, and healthcare administrators, regarding the perceived value and challenges of the EPAs could inform future enhancements of the competency framework. Finally, future studies could work on creating standardized assessment tools specifically designed for evaluating resident performance in relation to the identified EPAs, ensuring consistency and reliability across different training institutions. By addressing these areas, future studies can contribute to refining the educational framework for clinical microbiology and enhance the training of future professionals in the field.

## Conclusion

This study provides a validated set of 16 EPA items for clinical microbiology residency programs, addressing a significant gap in competency-based education for this specialty. These EPAs represent a standardized framework for assessing resident proficiency and guiding curriculum development. Addressing the challenges associated with EPAs that have lower agreement scores will further refine the framework, making it more adaptable and effective in diverse educational settings. Additionally, incorporating feedback mechanisms allows for continuous refinement of the EPA framework based on learner outcomes and evolving clinical practices.

## Electronic supplementary material

Below is the link to the electronic supplementary material.

Supplementary Material 1

## Data Availability

No datasets were generated or analysed during the current study.

## Abbreviations

EPAs: Entrustable professional activities
CBME: Competency-based medical education
QUEPA: Quality of entrustable professional activities
EQual: Queen’s epa quality rubric
CVI: Content validity index
ICC: Intraclass correlation coefficient
PGY1: First postgraduate year
PGY2: Second postgraduate year
PGY3: Third postgraduate year
