# Supplementary material for: Development of entrustable professional activities framework for clinical microbiology residency: a national multi-step consensus using modified Delphi study
Source: BMC Med Educ. 2025 May 22;25:755. doi: 10.1186/s12909-025-07345-x (PMC12100941; doi:10.1186/s12909-025-07345-x)
Supplement: Supplementary file 1 — Supplementary Material 1 [file 12909_2025_7345_MOESM1_ESM.pdf]

Appendix 1

Results of the phase 1 and Phase 2: Creation of the preliminary draft of 43 EPAs.

EPA – 1	Provide guidance for the resolution of preanalytical testing issues
**EPA – 2**	**Perform & interpret various staining techniques. *****
EPA – 3	Perform & interpret the motility of bacteria by hanging drop preparation of clinical specimens.
EPA – 4	Maintain both bacterial & fungal stock cultures.
EPA – 5	Carry out antibiotic sensitivity testing as per standard guidelines.
EPA – 6	Identify the pathogenic bacteria by aerobic and anaerobic culture methods.
EPA – 7	Perform & confirm the identification of fungus by routine fungal culture of clinical specimens.
EPA – 8	Perform and interpret rapid serological tests for bacterial and viral infection
EPA – 9	Perform diagnostic tests using automated machines
EPA – 10	Perform Molecular techniques for diagnosing various infectious diseases.
EPA – 11	able to manage needle stick injury documentation and care.
EPA - 12	Instruct the technician for handling & disposal of biomedical wastes
EPA - 13	aware & able to implement Infection control practices
***EPA – 14**	**Perform various other serology techniques like ELISA, and IFA.**
**EPA – 15**	**prepare a protocol for investigating any outbreak in the area *****
EPA – 16	Carry out systematic research work
EPA – 17	Perform as a team worker/leader.
EPA – 18	Teach microbiology to undergraduate medical students
EPA – 19	Advise on infection control measures
***EPA – 20**	**Ensure compliance with relevant legislative and regulatory frameworks**
EPA – 21	Participate in institutional infection control committee activities, e.g. audits, meetings
EPA – 22	advice on infection prevention, control, and immunization
EPA – 23	Implement, support, and develop procedures for safe laboratory practice
EPA – 24	Liaise between laboratory practice and infection control requirements, e.g. outbreak surveillance, subtyping
EPA – 25	Prepare articles to be sterilized by various and most appropriate methods
EPA – 26	Operate an autoclave safely and effectively
EPA – 27	Carry out various methods of the sterilization process.
EPA – 28	Detect faults in heat sterilizing apparatus
EPA – 29	Provide clinical leadership and support to the laboratory.
EPA – 30	Use the laboratory service effectively in the investigation, diagnosis, and management of infection.
EPA – 31	Manage and advise on important clinical syndromes where the infection is in the differential diagnosis.
EPA – 32	Lead and advise on treatment with and stewardship of antimicrobials.
EPA – 33	Provide continuity of care to inpatients and outpatients with suspected or proven infections.
EPA – 34	Manage and advise on imported infections.
**EPA – 35**	**Compose a diagnostic report for clinical laboratory testing *****

**EPA – 36**	**Evaluate and report critical values in the clinical laboratory (CP)*** **
**EPA – 37**	**Optimize test utilization (AP/CP laboratory management)*** **
EPA – 38	Evaluate and choose a new test or instrument (AP/CP laboratory management)
EPA – 39	Implement a new assay or test system (AP/CP laboratory management)
EPA – 40	Improve quality and patient safety (AP/CP laboratory management)
EPA - 41	Perform a laboratory accreditation inspection (AP/CP laboratory management)
***EPA – 42**	**Familiar with norms & requirements of NABL, NABH accreditation**
***EPA - 43**	**Perform and validate quality control procedures for newly received culture media and reagents**

*The following EPAs were excluded based on EQual rubric evaluation:

EPA – 14 Perform various other serology techniques like ELISA, and IFA.

EPA – 20 Ensure compliance with relevant legislative and regulatory frameworks

EPA – 42 Familiar with norms & requirements of NABL, NABH accreditation

EPA – 43 Perform and validate quality control procedures for newly received culture media and reagents.

These EPAs scored below the predefined cutoff score of 4.07, indicating they did not meet the criteria for alignment with EPA standards.

*** **The EPAs 2, 15, 35, 36 and 37** generated in Phases 1 and 2 (Appendix 1) underwent qualitative rephrasing to enhance their clarity, specificity, and relevance to the context of clinical microbiology practice in Egypt.

EPA-2, 'Perform & interpret various staining techniques,' was revised in Phase 3 to become 'Perform & interpret various staining techniques like gram staining, Acid-fast staining, negative staining, and special staining.

EPA-15, initially 'prepare a protocol for investigating any outbreak in the area' was changed to 'prepare a protocol for investigating any outbreak in the area like cholera, typhoid, brucellosis, and viral infections.

EPA -35 'Compose a diagnostic report for clinical laboratory testing', was rephrased to 'Compose a diagnostic report for clinical laboratory testing requiring pathologist interpretation'.

EPA – 36 Evaluate and report critical values in the clinical laboratory was rephrased to Identity, Evaluate, report, and communicate critical value and clinically urgent results.

EPA – 37 Optimize test utilization (AP/CP laboratory management) was rephrased to EPA37: Optimize test utilization in the Clinical Microbiology Laboratory

Appendix 2

Round 1: Modified Delphi Technique survey

Development of Entrustable Professional Activities for Clinical Microbiology Residency: A national consensus using modified Delphi Study

Dear Expert, Kindly be informed that you have been selected as a subject matter expert to review this list of activities and identify (in your professional opinion) to what degree these skills are essential for demonstrating competency in Clinical Microbiology.

I wonder if it is important to provide the survey respondent to provide additional commentary on any skills that are not listed in my survey.

This form was developed to provide a standardized and validated list of Entrustable Professional Activities for Clinical Microbiology Residency.

The data of this form will be used in research, and you will be acknowledged for your contribution. If you would like to participate, please review the questionnaire using the following evaluation form.

A. General demographic questions

Name
Email
Department
Academic rank
Years of experience in microbiology
University

B. EPA-specific questions

Per individual EPA, you will be asked to rate whether this EPA reflects an essential core task **for a Clinical Microbiology Residency** physician (indispensability) and whether or not the description of this EPA is sufficiently clear (clarity)

EPA 1: Provide guidance for the resolution of preanalytical testing issues

a. *To what extent does this EPA describe an essential task for a* **Clinical Microbiology Residency** physician

None	***Low***	***Medium***	***High***	***Very high***

b. *The clarity of the description of this EPA is:*

Very poor	***Poor***	***Neither poor nor good***	***Good***	***Very good***

c. *Additional comments for this EPA*

EPA 2: perform & interpret various staining techniques like gram staining. Acid-fast staining. negative staining and special staining ***

a. *To what extent does this EPA describe an essential task for a* **Clinical Microbiology Residency** physician

None	***Low***	***Medium***	***High***	***Very high***

b. *The clarity of the description of this EPA is:*

Very poor	***Poor***	***Neither poor nor good***	***Good***	***Very good***

c. *Additional comments for this EPA*

EPA 3: perform & interpret the motility of bacteria by hanging drop preparation of clinical specimens.

a. *To what extent does this EPA describe an essential task for a* **Clinical Microbiology Residency** physician

**None**	**Low**	**Medium**	**High**	**Very high**

b. *The clarity of the description of this EPA is:*

**Very poor**	**Poor**	**Neither poor nor good**	**Good**	**Very good**

c. *dditional comments for this EPA*

EPA 4: maintain both bacterial & fungal stock cultures.

a. *To what extent does this EPA describe an essential task for a* **Clinical Microbiology Residency** physician

None	***Low***	***Medium***	***High***	***Very high***

b. *The clarity of the description of this EPA is:*

Very poor	***Poor***	***Neither poor nor good***	***Good***	***Very good***

c. *Additional comments for this EPA*

EPA 5: Carry out antibiotic sensitivity testing as per Current standard guidelines.

a. *To what extent does this EPA describe an essential task for a* **Clinical Microbiology Residency** physician

none	***Low***	***Medium***	***High***	***Very high***

b. *The clarity of the description of this EPA is:*

Very poor	***Poor***	***Neither poor nor good***	***Good***	***Very good***

c. *Additional comments for this EPA*

EPA 6: identify the pathogenic bacteria by aerobic and anaerobic culture methods.

a. *To what extent does this EPA describe an essential task for a* **Clinical Microbiology Residency** physician

none	***Low***	***Medium***	***High***	***Very high***

b. *The clarity of the description of this EPA is:*

Very poor	***Poor***	***Neither poor nor good***	***Good***	***Very good***

c. *Additional comments for this EPA*

EPA 7: perform & confirm the identification of fungus by routine fungal.

a. *To what extent does this EPA describe an essential task for a* **Clinical Microbiology Residency** physician

none	***Low***	***Medium***	***High***	***Very high***

b. *The clarity of the description of this EPA is:*

Very poor	***Poor***	***Neither poor nor good***	***Good***	***Very good***

c. *Additional comments for this EPA*

EPA 8: perform and interpret rapid serological tests for bacterial and viral

infection

a. *To what extent does this EPA describe an essential task for a* **Clinical Microbiology Residency** physician

None	***Low***	***Medium***	***High***	***Very high***

b. *The clarity of the description of this EPA is:*

Very poor	***Poor***	***Neither poor nor good***	***Good***	***Very good***

c. *Additional comments for this EPA*

EPA 9: perform diagnostic tests using automated machines.

a. *To what extent does this EPA describe an essential task for a* **Clinical Microbiology Residency** physician

None	***Low***	***Medium***	***High***	***Very high***

b. *The clarity of the description of this EPA is:*

Very poor	***Poor***	***Neither poor nor good***	***Good***	***Very good***

c. *Additional comments for this EPA*

EPA 10: perform Molecular techniques for diagnosing various infectious diseases.

a. *To what extent does this EPA describe an essential task for a* **Clinical Microbiology Residency** physician

None	***Low***	***Medium***	***High***	***Very high***

b. *The clarity of the description of this EPA is:*

Very poor	***Poor***	***Neither poor nor good***	***Good***	***Very good***

c. *Additional comments for this EPA*

EPA 11: able to manage needle stick injury documentation and care.

a. *To what extent does this EPA describe an essential task for a* **Clinical Microbiology Residency** physician

None	***Low***	***Medium***	***High***	***Very high***

b. *The clarity of the description of this EPA is:*

Very poor	***Poor***	***Neither poor nor good***	***Good***	***Very good***

c. *Additional comments for this EPA*

EPA 12: instruct the technician for handling & disposal of biomedical wastes.

a. *To what extent does this EPA describe an essential task for a* **Clinical Microbiology Residency** physician

None	***Low***	***Medium***	***High***	***Very high***

b. *The clarity of the description of this EPA is:*

Very poor	***Poor***	***Neither poor nor good***	***Good***	***Very good***

c. *Additional comments for this EPA*

EPA 13: aware & able to implement Infection control practices.

a. *To what extent does this EPA describe an essential task for a* **Clinical Microbiology Residency** physician

None	***Low***	***Medium***	***High***	***Very high***

b. *The clarity of the description of this EPA is:*

Very poor	***Poor***	***Neither poor nor good***	***Good***	***Very good***

c. *Additional comments for this EPA*

EPA 15 : prepare a protocol for investigating any outbreak in the area like cholera, typhoid, brucellosis, and viral infections. ***

a. *To what extent does this EPA describe an essential task for a* **Clinical Microbiology Residency** physician

none	***Low***	***Medium***	***High***	***Very high***

b. *The clarity of the description of this EPA is:*

Very poor	***Poor***	***Neither poor nor good***	***Good***	***Very good***

c. *Additional comments for this EPA*

EPA 16 Carry out systematic research work.

a. *To what extent does this EPA describe an essential task for a* **Clinical Microbiology Residency** physician

none	***Low***	***Medium***	***High***	***Very high***

b. *The clarity of the description of this EPA is:*

Very poor	***Poor***	***Neither poor nor good***	***Good***	***Very good***

c. *Additional comments for this EPA*

EPA 17: perform as a team worker/leader.

a. *To what extent does this EPA describe an essential task for a* **Clinical Microbiology Residency** physician

none	***Low***	***Medium***	***High***	***Very high***

b. *The clarity of the description of this EPA is:*

Very poor	***Poor***	***Neither poor nor good***	***Good***	***Very good***

c. *Additional comments for this EPA*

EPA 18: teach microbiology to undergraduate medical students.

a. *To what extent does this EPA describe an essential task for a* **Clinical Microbiology Residency** physician

none	***Low***	***Medium***	***High***	***Very high***

b. *The clarity of the description of this EPA is:*

Very poor	***Poor***	***Neither poor nor good***	***Good***	***Very good***

c. *Additional comments for this EPA*

EPA 19: Advise on infection control measures to patients, colleagues, and institutional bodies.

a. *To what extent does this EPA describe an essential task for a* **Clinical Microbiology Residency** physician

none	***Low***	***Medium***	***High***	***Very high***

b. *The clarity of the description of this EPA is:*

Very poor	***Poor***	***Neither poor nor good***	***Good***	***Very good***

c. *Additional comments for this EPA*

EPA 21: Participate in institutional infection control committee activities, e.g. audits, and meetings.

a. *To what extent does this EPA describe an essential task for a* **Clinical Microbiology Residency** physician

none	***Low***	***Medium***	***High***	***Very high***

b. *The clarity of the description of this EPA is:*

Very poor	Poor	Neither poor nor good	Good	Very good

c. *Additional comments for this EPA*

EPA 22: advice on infection prevention, control, and immunization

To what extent does this EPA describe an essential task for a **Clinical Microbiology Residency** physician

none	Low	Medium	High	Very high

a. *The clarity of the description of this EPA is:*

Very poor	Poor	Neither poor nor good	Good	Very good

b. *Additional comments for this EPA*

EPA 23: Implement, support, and develop procedures for safe laboratory practice.

a. *To what extent does this EPA describe an essential task for a* **Clinical Microbiology Residency** physician

none	Low	Medium	High	Very high

b. *The clarity of the description of this EPA is:*

Very poor	Poor	Neither poor nor good	Good	Very good

c. *Additional comments for this*

EPA 24 : Liaise between laboratory practice and infection control requirements, e.g. outbreak surveillance, subtyping

a. *To what extent does this EPA describe an essential task for a* **Clinical Microbiology Residency** physician

none	Low	Medium	High	Very high

b. *The clarity of the description of this EPA is:*

Very poor	Poor	Neither poor nor good	Good	Very good

c. *Additional comments for this EPA*

EPA 25: Prepare articles to be sterilized by various and most appropriate methods.

a. *To what extent does this EPA describe an essential task for a* **Clinical Microbiology Residency** physician

none	Low	Medium	High	Very high

b. *The clarity of the description of this EPA is:*

Very poor	Poor	Neither poor nor good	Good	Very good

c. *Additional comments for this EPA*

EPA 26: Operate an autoclave safely and effectively.

To what extent does this EPA describe an essential task for a **Clinical Microbiology Residency** physician

none	Low	Medium	High	Very high

a. *The clarity of the description of this EPA is:*

Very poor	Poor	Neither poor nor good	Good	Very good

b. *Additional comments for this EPA*

EPA 27: Carry out various methods of the sterilization process.

a. *To what extent does this EPA describe an essential task for a* **Clinical Microbiology Residency** physician

none	Low	Medium	High	Very high

b. *The clarity of the description of this EPA is:*

Very poor	Poor	Neither poor nor good	Good	Very good

c. *Additional comments for this EPA*

EPA 28: Detect faults in heat sterilizing apparatus.

a. *To what extent does this EPA describe an essential task for a* **Clinical Microbiology Residency** physician

none	Low	Medium	High	Very high

b. *The clarity of the description of this EPA is:*

Very poor	***Poor***	***Neither poor nor good***	***Good***	***Very good***

c. *Additional comments for this EPA*

EPA 29: provide clinical leadership and support to the laboratory.

a. *To what extent does this EPA describe an essential task for a* **Clinical Microbiology Residency** physician

none	***Low***	***Medium***	***High***	***Very high***

b. *The clarity of the description of this EPA is:*

Very poor	***Poor***	***Neither poor nor good***	***Good***	***Very good***

c. *Additional comments for this EPA*

EPA 30 – use the laboratory service effectively in the investigation, diagnosis, and management of infection.

a. *To what extent does this EPA describe an essential task for a* **Clinical Microbiology Residency** physician

none	***Low***	***Medium***	***High***	***Very high***

b. *The clarity of the description of this EPA is:*

Very poor	***Poor***	***Neither poor nor good***	***Good***	***Very good***

c. *Additional comments for this EPA*

EPA 31 - manage and advise on important clinical syndromes where the infection is in the differential diagnosis.

a. *To what extent does this EPA describe an essential task for a* **Clinical Microbiology Residency** physician

none	***Low***	***Medium***	***High***	***Very high***

b. *The clarity of the description of this EPA is:*

Very poor	***Poor***	***Neither poor nor good***	***Good***	***Very good***

c. *Additional comments for this EPA*

EPA 32 - lead and advise on treatment with and stewardship of antimicrobials

To what extent does this EPA describe an essential task for a **Clinical Microbiology Residency** physician

none	***Low***	***Medium***	***High***	***Very high***

a. *The clarity of the description of this EPA is:*

Very poor	***Poor***	***Neither poor nor good***	***Good***	***Very good***

b. *Additional comments for this EPA*

EPA 33 - provide continuity of care to inpatients and outpatients with suspected or proven infections.

To what extent does this EPA describe an essential task for a **Clinical Microbiology Residency** physician

none	***Low***	***Medium***	***High***	***Very high***

a. *The clarity of the description of this EPA is:*

Very poor	***Poor***	***Neither poor nor good***	***Good***	***Very good***

b. *Additional comments for this EPA*

EPA 34- manage and advise on imported infections.

a. *To what extent does this EPA describe an essential task for a* **Clinical Microbiology Residency** physician

none	***Low***	***Medium***	***High***	***Very high***

b. *The clarity of the description of this EPA is:*

Very poor	***Poor***	***Neither poor nor good***	***Good***	***Very good***

c. *Additional comments for this EPA*

EPA 35 - Compose a diagnostic report for clinical laboratory testing requiring pathologist interpretation (CP)***

To what extent does this EPA describe an essential task for a **Clinical Microbiology Residency** *To what extent does this EPA describe an essential task for a* **Clinical Microbiology Residency** physician

none	***Low***	***Medium***	***High***	***Very high***

a. *The clarity of the description of this EPA is:*

Very poor	***Poor***	***Neither poor nor good***	***Good***	***Very good***

b. *Additional comments for this EPA*

EPA 36 - Evaluate, report, and communicate critical value and clinically urgent results.

a. *To what extent does this EPA describe an essential task for a* **Clinical Microbiology Residency** physician

none	***Low***	***Medium***	***High***	***Very high***

b. *The clarity of the description of this EPA is:*

Very poor	***Poor***	***Neither poor nor good***	***Good***	***Very good***

c. *Additional comments for this EPA*

EPA 37 - Optimize test utilization in the clinical microbiology laboratory

a. *To what extent does this EPA describe an essential task for a* **Clinical Microbiology Residency** physician

none	***Low***	***Medium***	***High***	***Very high***

b. *The clarity of the description of this EPA is:*

Very poor	***Poor***	***Neither poor nor good***	***Good***	***Very good***

c. *Additional comments for this EPA*

EPA 38 - Evaluate and choose a new test or instrument (AP/CP laboratory management)

a. *To what extent does this EPA describe an essential task for a* **Clinical Microbiology Residency** physician

none	***Low***	***Medium***	***High***	***Very high***

b. *The clarity of the description of this EPA is:*

Very poor	***Poor***	***Neither poor nor good***	***Good***	***Very good***

c. *Additional comments for this EPA*

EPA 39 - Implement a new assay or test system (AP/CP laboratory management)
To what extent does this EPA describe an essential task for a **Clinical Microbiology Residency** physician

none	***Low***	***Medium***	***High***	***Very high***

a. *The clarity of the description of this EPA is:*

Very poor	***Poor***	***Neither poor nor good***	***Good***	***Very good***

b. *Additional comments for this EPA*

EPA 40- Improve quality and patient safety (AP/CP laboratory management)

To what extent does this EPA describe an essential task for a **Clinical Microbiology Residency** physician

none	***Low***	***Medium***	***High***	***Very high***

a. *The clarity of the description of this EPA is:*

Very poor	***Poor***	***Neither poor nor good***	***Good***	***Very good***

b. *Additional comments for this EPA*

EPA 41 - Perform a laboratory accreditation inspection (AP/CP laboratory management)

a. *To what extent does this EPA describe an essential task for a* **Clinical Microbiology Residency** physician

none	***Low***	***Medium***	***High***	***Very high***

b. *The clarity of the description of this EPA is:*

Very poor	***Poor***	***Neither poor nor good***	***Good***	***Very good***

c. *Additional comments for this EPA*

Appendix 3

Round 2: Modified Delphi Technique survey

Development of Entrustable Professional Activities for Clinical Microbiology Residency: A National Consensus Using Modified Delphi Study: 2nd round

Dear Expert, Kindly be informed that you have been selected as a subject matter expert to determine the year of entrustment for each EPA. A broad consensus among different stakeholders of clinical microbiology is helpful in facilitating implementation.

I wonder if it is important to provide the survey respondent to provide additional comments and feedback for each EPA.

This form was developed to provide a standardized and validated year of entrustment for Clinical Microbiology Residency.

The data of this form will be used in research, and you will be acknowledged for your contribution. If you would like to participate, please review the questionnaire using the following evaluation form.

EPA definition and concepts

Definition: Entrustable professional activity is a unit of professional practice that can be fully entrusted to a trainee, once he or she has demonstrated the necessary competence to execute this activity unsupervised. "Unit" signifies a discrete task (e.g." Managing patients with cataract") or bundle of tasks (e.g." Performing the procedures of internal medicine "or" Managing an inpatient medical service") and should be suitable for credentialing.

They are **observable**, **measurable**, and **executable** within a **specific time frame.** EPAs use **observable work descriptors.**

<u>Five main levels of entrustment have been described:</u>

Level 1: The learner is allowed to be **present and observe,** not to perform an EPA;
Level 2: The learner is allowed to execute the EPA with **direct, proactive supervision,** present in the laboratory ;
Level 3: The learner is allowed to carry out the EPA **without a supervisor in the laboratory, but quickly available** if needed, i.e., with indirect, reactive, supervision;
Level 4: The learner is allowed to work **unsupervised**;
Level 5: The learner is allowed to **provide supervision** to more junior learners

General demographic questions

Name
Email
Department
Academic rank
Years of experience in microbiology
University

The current list of 15 EPAs has sufficient content validation

1. **EPA 1: Provide guidance for the resolution of preanalytical testing issues**
2. **EPA 2: Perform & interpret various staining techniques like gram staining. Acid-fast staining. negative staining and special staining**
3. **EPA 5: Carry out antibiotic sensitivity testing as per Current standard guidelines.**
4. **EPA6: Identify the pathogenic bacteria by aerobic and anaerobic culture methods.**
5. **EPA 11: able to manage needle stick injury documentation and care.**
6. **EPA 12: Instruct the technician for handling & disposal of biomedical wastes.**
7. **EPA 13: aware & able to implement Infection control practices.**
8. **EPA 16 Carry out systematic research work.**
9. **EPA 17: Perform as a team worker/leader.**
10. **EPA 19: Advice on infection control measures**
11. **EPA 23: Implement, support, and develop procedures for safe laboratory practice.**
12. **EPA 30: Use the laboratory service effectively in the investigation, diagnosis, and management of infection.**
13. **EPA 35: Compose a diagnostic report for clinical laboratory testing requiring pathologist interpretation**
14. **EPA 36: Identity, Evaluate, report, and communicate critical value and clinically urgent results**
15. **EPA 37 - Optimize test utilization in the clinical microbiology laboratory**

The current list of 4 EPAs has insufficient content validation and need to be revised
EPA 7: perform & confirm the identification of fungus by routine fungal
EPA 8: perform and interpret rapid serological tests for bacterial and viral infection
EPA 9: perform diagnostic tests using automated machines.
EPA 29: provide clinical leadership and support to the laboratory.

EPA 7: perform & confirm the identification of fungus by routine fungal

a. *To what extent does this EPA describe an essential task for a* **Clinical Microbiology Residency** physician

none	***Low***	***Medium***	***High***	***Very high***

b. *The clarity of the description of this EPA is:*

**Very poor**	**Poor**	**Neither poor nor good**	**Good**	**Very good**

c. *Additional comments for this EPA*

EPA 8: perform and interpret rapid serological tests for bacterial and viral infection
To what extent does this EPA describe an essential task for a **Clinical Microbiology Residency** physician

none	***Low***	***Medium***	***High***	***Very high***

a. *The clarity of the description of this EPA is:*

**Very poor**	**Poor**	**Neither poor nor good**	**Good**	**Very good**

b. *Additional comments for this EPA*

EPA 9: perform diagnostic tests using automated machines.
To what extent does this EPA describe an essential task for a **Clinical Microbiology Residency** physician

none	***Low***	***Medium***	***High***	***Very high***

a. *The clarity of the description of this EPA is:*

**Very poor**	**Poor**	**Neither poor nor good**	**Good**	**Very good**

b. *Additional comments for this EPA*

EPA 29: provide clinical leadership and support to the laboratory.

a. *To what extent does this EPA describe an essential task for a* **Clinical Microbiology Residency** physician

none	***Low***	***Medium***	***High***	***Very high***

c. *The clarity of the description of this EPA is:*

Very poor	Poor	Neither poor nor good	Good	Very good

d. *Additional comments for this EPA*

Section 9 -The resident is expected to reach Level 4 (ready for unsupervised practice):
Level 1: the learner is allowed to be present and observe, not to enact an EPA
Level 2: the learner is allowed to execute the EPA with direct, pro-active supervision, present in the room
Level 3: the learner is allowed to carry out the EPA without a supervisor in the room, but quickly available if needed, i.e. with indirect, reactive, supervision
Level 4: the learner is allowed to work unsupervised
Level 5: the learner is allowed to provide supervision to more junior learners.

Section 10 - EPA 1: Provide guidance for the resolution of preanalytical testing issues
The resident is expected to reach Level 4 (ready for unsupervised practice)

post graduate year -1	post graduate year -2	post graduate year -3

Section 11 - EPA 2: Perform & interpret various staining techniques like gram staining. Acid-fast staining. negative staining and special staining
The resident is expected to reach Level 4 (ready for unsupervised practice)

post graduate year -1	post graduate year -2	post graduate year -3

Section 12 - EPA 5: Carry out antibiotic sensitivity testing as per Current standard guidelines.
The resident is expected to reach Level 4 (ready for unsupervised practice)

post graduate year -1	post graduate year -2	post graduate year -3

Section 13 - EPA6: Identify the pathogenic bacteria by aerobic and anaerobic culture methods The resident is expected to reach Level 4 (ready for unsupervised practice)

post graduate year -1	post graduate year -2	post graduate year -3

Section 14- EPA 11: able to manage needle stick injury documentation and care.
The resident is expected to reach Level 4 (ready for unsupervised practice)

post graduate year -1	post graduate year -2	post graduate year -3

Section 15 - EPA 12: Instruct the technician for handling & disposal of biomedical wastes.
The resident is expected to reach Level 4 (ready for unsupervised practice)

post graduate year -1	post graduate year -2	post graduate year -3

Section 16- EPA 13: aware & able to implement Infection control practices.
The resident is expected to reach Level 4 (ready for unsupervised practice)

post graduate year -1	post graduate year -2	post graduate year -3

Section 17 - EPA 16 Carry out systematic research work.
The resident is expected to reach Level 4 (ready for unsupervised practice)

post graduate year -1	post graduate year -2	post graduate year -3

Section 18 - EPA 17: Perform as a team worker/leader.
The resident is expected to reach Level 4 (ready for unsupervised practice)

post graduate year -1	post graduate year -2	post graduate year -3

Section 19 - EPA 19: Advice on infection control measures
The resident is expected to reach Level 4 (ready for unsupervised practice)

post graduate year -1	post graduate year -2	post graduate year -3

Section 20 - EPA 23: Implement, support, and develop procedures for safe laboratory practice.
The resident is expected to reach Level 4 (ready for unsupervised practice)

post graduate year -1	post graduate year -2	post graduate year -3

Section 21- EPA 29: provide clinical leadership and support to the laboratory.
The resident is expected to reach Level 4 (ready for unsupervised practice)

post graduate year -1	post graduate year -2	post graduate year -3

Section 22- EPA 30: Use the laboratory service effectively in the investigation, diagnosis, and management of infection.

The resident is expected to reach Level 4 (ready for unsupervised practice)

post graduate year -1	post graduate year -2	post graduate year -3

Section 23 - EPA 35: Compose a diagnostic report for clinical laboratory testing requiring pathologist interpretation

The resident is expected to reach Level 4 (ready for unsupervised practice)

post graduate year -1	post graduate year -2	post graduate year -3

Section 24 - EPA 36: Identity, Evaluate, report, and communicate critical value and clinically urgent results The resident is expected to reach Level 4 (ready for unsupervised practice)

post graduate year -1	post graduate year -2	post graduate year -3

Section 25- EPA 37 - Optimize test utilization clinical microbiology laboratory

The resident is expected to reach Level 4 (ready for unsupervised practice)

post graduate year -1	post graduate year -2	post graduate year -3

Appendix 4

Round 3: Modified Delphi Technique survey

Section 1 - Development of Entrustable Professional Activities for Clinical Microbiology Residency: A national consensus using modified Delphi Study: 3rd round

Dear Expert, Kindly be informed that you have been selected as a subject matter expert to review this list of activities and identify (in your professional opinion) your agreement

I wonder if it is important to provide the survey respondent to provide additional commentary on any skills that are not listed in my survey.

This form was developed to provide a standardized and validated list of Entrustable Professional Activities for Clinical Microbiology Residency.

The data of this form will be used in research, and you will be acknowledged for your contribution. If you would like to participate, please review the questionnaire using the following evaluation form.

Section2 - General demographic questions

Name

Email

Department

Academic rank

Years of experience in microbiology

University

Section 10 - EPA 1: Provide guidance for the resolution of preanalytical testing issues

**YES**	**NO**

Section 11 - EPA 2: Perform & interpret various staining techniques like gram staining. Acid-fast staining. negative staining and special staining

**YES**	**NO**

Section 12 - EPA 5: Carry out antibiotic sensitivity testing as per Current standard guidelines.

**YES**	**NO**

Section 13 - EPA6: Identify the pathogenic bacteria by aerobic and anaerobic culture methods

**YES**	**NO**

Section 14- EPA 11: able to manage needle stick injury documentation and care.

**YES**	**NO**

Section 15 - EPA 12: Instruct the technician for handling & disposal of biomedical wastes.

**YES**	**NO**

Section 16- EPA 13: aware & able to implement Infection control practices.

**YES**	**NO**

Section 17 - EPA 16 Carry out systematic research work.

**YES**	**NO**

Section 18 - EPA 17: Perform as a team worker/leader.

**YES**	**NO**

Section 19 - EPA 19: Advice on infection control measures

**YES**	**NO**

Section 20 - EPA 23: Implement, support, and develop procedures for safe laboratory practice.

**YES**	**NO**

Section 21- EPA 29: provide clinical leadership and support to the laboratory

**YES**	**NO**

Section 22- EPA 30: Use the laboratory service effectively in the investigation, diagnosis, and management of infection.

**YES**	**NO**

Section 23 - EPA 35: Compose a diagnostic report for clinical laboratory testing requiring pathologist interpretation

**YES**	**NO**

Section 24- EPA 36: Identity, Evaluate, report, and communicate critical value and clinically urgent results

**YES**	**NO**

Section 25- EPA 37 - Optimize test utilization clinical microbiology laboratory

**YES**	**NO**

Appendix 5

Proposed ordered EPA List

Technical & Diagnostic EPAs:

1. **Perform & interpret various staining techniques like gram staining, Acid-fast staining, negative staining, and special staining (EPA 2):** Foundation for microscopic diagnosis.
2. **Carry out antibiotic sensitivity testing as per Current standard guidelines (EPA 3):** Essential for antimicrobial stewardship.
3. **Identify the pathogenic bacteria by aerobic and anaerobic culture methods (EPA 4):** Core competency in bacterial identification.
4. **Compose a diagnostic report for clinical laboratory testing requiring pathologist interpretation (EPA 14):** Crucial for communicating findings.
5. **Identity, Evaluate, report, and communicate critical value and clinically urgent results (EPA 15):** Time sensitivity and proper documentation.

Laboratory Management & Safety EPAs:

6. **Provide guidance for the resolution of preanalytical testing issues (EPA 1):** Ensures sample integrity.
7. **Implement, support, and develop procedures for safe laboratory practice (EPA 11):** Prioritizes safety.
8. **Instruct the technician for handling & disposal of biomedical wastes (EPA 6):** Protects lab environment and staff.
9. **Able to manage needle stick injury documentation and care (EPA 5):** Manages risks of lab personnel.
10. **Optimize test utilization (clinical pathology laboratory management) (EPA 16):** Clinical pathology laboratory management.

Infection Control & Antimicrobial Stewardship EPAs:

11. **Use the laboratory service effectively in the investigation, diagnosis, and management of infection (EPA 13):** Combines diagnostic and interpretive skills.
12. **Aware & able to implement Infection control practices (EPA 7):** Prevents spread of infectious agents.
13. **Advice on infection control measures (EPA 10):** Provides expertise to clinical teams.

Leadership & Communication EPAs:

14. **Perform as a team worker/leader (EPA 9):** Promotes team collaboration.
15. **Provide clinical leadership and support to the laboratory (EPA 12):** Provides clinical leadership skills.
16. **Carry out systematic research work (EPA 8):** Incorporates research aspects.
